# Age-dependent dysregulation of collagen metabolism in primary cardiac fibroblasts *in vitro*

**DOI:** 10.1093/cvr/cvag093

**Published:** 2026-04-25

**Authors:** Kalyani Pandya, Carlos J Alcaide-Corral, Timaeus E F Morgan, Kerry O’Rourke, Leanne M Riley, Islay Cranston, Andrew Sutherland, Mark G MacAskill, Gillian A Gray, Adriana A S Tavares

**Affiliations:** Centre for Cardiovascular Science, The University of Edinburgh, Queens Medical Research Institute, 47 Little France Crescent, Edinburgh EH16 4TJ, UK; Centre for Cardiovascular Science, The University of Edinburgh, Queens Medical Research Institute, 47 Little France Crescent, Edinburgh EH16 4TJ, UK; Edinburgh Imaging, The University of Edinburgh, Edinburgh EH16 4TJ, UK; Centre for Cardiovascular Science, The University of Edinburgh, Queens Medical Research Institute, 47 Little France Crescent, Edinburgh EH16 4TJ, UK; Edinburgh Imaging, The University of Edinburgh, Edinburgh EH16 4TJ, UK; Centre for Cardiovascular Science, The University of Edinburgh, Queens Medical Research Institute, 47 Little France Crescent, Edinburgh EH16 4TJ, UK; Edinburgh Imaging, The University of Edinburgh, Edinburgh EH16 4TJ, UK; School of Chemistry, University of Glasgow, Glasgow G12 8QQ, UK; Centre for Cardiovascular Science, The University of Edinburgh, Queens Medical Research Institute, 47 Little France Crescent, Edinburgh EH16 4TJ, UK; Edinburgh Imaging, The University of Edinburgh, Edinburgh EH16 4TJ, UK; School of Chemistry, University of Glasgow, Glasgow G12 8QQ, UK; Centre for Cardiovascular Science, The University of Edinburgh, Queens Medical Research Institute, 47 Little France Crescent, Edinburgh EH16 4TJ, UK; Edinburgh Imaging, The University of Edinburgh, Edinburgh EH16 4TJ, UK; Centre for Cardiovascular Science, The University of Edinburgh, Queens Medical Research Institute, 47 Little France Crescent, Edinburgh EH16 4TJ, UK; Centre for Cardiovascular Science, The University of Edinburgh, Queens Medical Research Institute, 47 Little France Crescent, Edinburgh EH16 4TJ, UK; Edinburgh Imaging, The University of Edinburgh, Edinburgh EH16 4TJ, UK

**Keywords:** Cardiac fibroblast, Fibrosis, Ageing, Sex differences, Collagen, Fluoroproline

## Abstract

**Introduction:**

Fibroblast-driven collagen remodelling is a key process in cardiac ageing and fibrosis, yet how age and sex alter fibroblast collagen handling remains poorly characterized. This proof-of-concept study aimed to address this knowledge gap, as currently the effects of age and sex on cardiac fibroblast (cFb) collagen metabolism remain incompletely understood.

**Methods and results:**

cFbs were isolated from young (4-week) and aged (18-month) Sprague–Dawley rats and cultured *in vitro.* Unhydroxylated (immature) and hydroxylated (mature) collagen synthesis was estimated using *cis*- and *trans*-4-[^18^F]fluoro-L-proline PET radiotracers as a proxy measure of collagen synthesis. Insoluble and soluble collagen deposition was assessed using colorimetric assays. No sex differences were observed in hydroxylated and hydroxylated collagen synthesis. Ageing resulted in increased hydroxylated and unhydroxylated collagen synthesis in males. Insoluble collagen deposition was higher in young males than females and decreased with ageing.

**Conclusion:**

Collagen metabolism in cFbs is influenced by age, with ageing uncoupling synthesis and deposition. These findings provide preliminary mechanistic insight and intrinsic fibroblast-level differences that may underlie age- and sex-dependent cardiac remodelling.

**Time of primary review: 39 days**

## 1. Introduction

Cardiac fibroblasts (cFbs) regulate myocardial collagen synthesis and extracellular matrix (ECM) homeostasis, playing a central role in cardiac remodelling during ageing and disease.^1^ Age-related myocardial fibrosis is a hallmark of cardiovascular ageing, contributing to myocardial stiffness, diastolic dysfunction, and heart failure with preserved ejection fraction (HFpEF). However, fibroblast-intrinsic mechanisms driving these fibrotic changes, and how they differ by age and sex, remain unclear.^2^ Despite growing recognition of sex-specific differences in cardiac remodelling—often attributed to hormonal factors—the cellular and molecular basis, particularly at the level of collagen metabolism in cFbs, is poorly characterized. Proline hydroxylation stabilizes the collagen triple helix, while unhydroxylated collagen indicates impaired maturation; measuring both provides key insights into pathological ECM remodelling.^3^

This study assessed the influence of sex and ageing on cFbs collagen metabolism *in vitro*. Using primary cFbs isolated from young (4-week-old) and aged (18-month-old) rats, we estimated unhydroxylated and hydroxylated collagen synthesis with Positron Emission Tomography radiotracers *cis*- and *trans*-4-[^18^F]fluoro-L-proline as proxy measures.^3^ To evaluate ECM accumulation, we measured soluble and insoluble collagen deposition using colorimetric assays. This work is relevant given growing interest in fibroblast-targeted therapies and the burden of age-related cardiovascular fibrosis.^4^

## 2. Methods

Animal procedures followed UK Animals (Scientific Procedures) Act 1986 and EU Directive 2010/63/EU, with approval from the University of Edinburgh Animal Welfare and Ethical Review Committee. Sprague-Dawley rats (Charles River, Tranent) were housed under standard conditions, anaesthetized with 5% isoflurane, and euthanized by cervical dislocation per institutional/national guidelines.

Primary cFbs were isolated from rat ventricles and cultured under standardized conditions.^5^ Cell identity was confirmed by immunocytochemistry for αSMA, vimentin and PDGFRα (passages 2–4). Heterogeneous αSMA expression indicated mixed activation states. Radiolabelled *cis*-/*trans*-4-[^18^F]fluoro-L-proline—were synthesized using optimized protocols.^6^ cFbs were incubated with tracers for 90 min (Td: *Cis* 52 min; *trans* 30 min); uptake was quantified and normalized to total protein. Tracer uptake was interpreted as a proxy of collagen-related metabolism. Soluble and insoluble collagen deposition was assessed using picrosirius red dye based Sircol™ assays (Biocolor, UK). Sex-dependent differences were evaluated in young animals only. Data are shown as mean ± SEM with individual points. Normality was assessed by Shapiro–Wilk; data were analysed using Mann–Whitney *U* or unpaired *t*-tests as appropriate.

## 3. Results

### 3.1 Collagen synthesis

**Figure 1:**
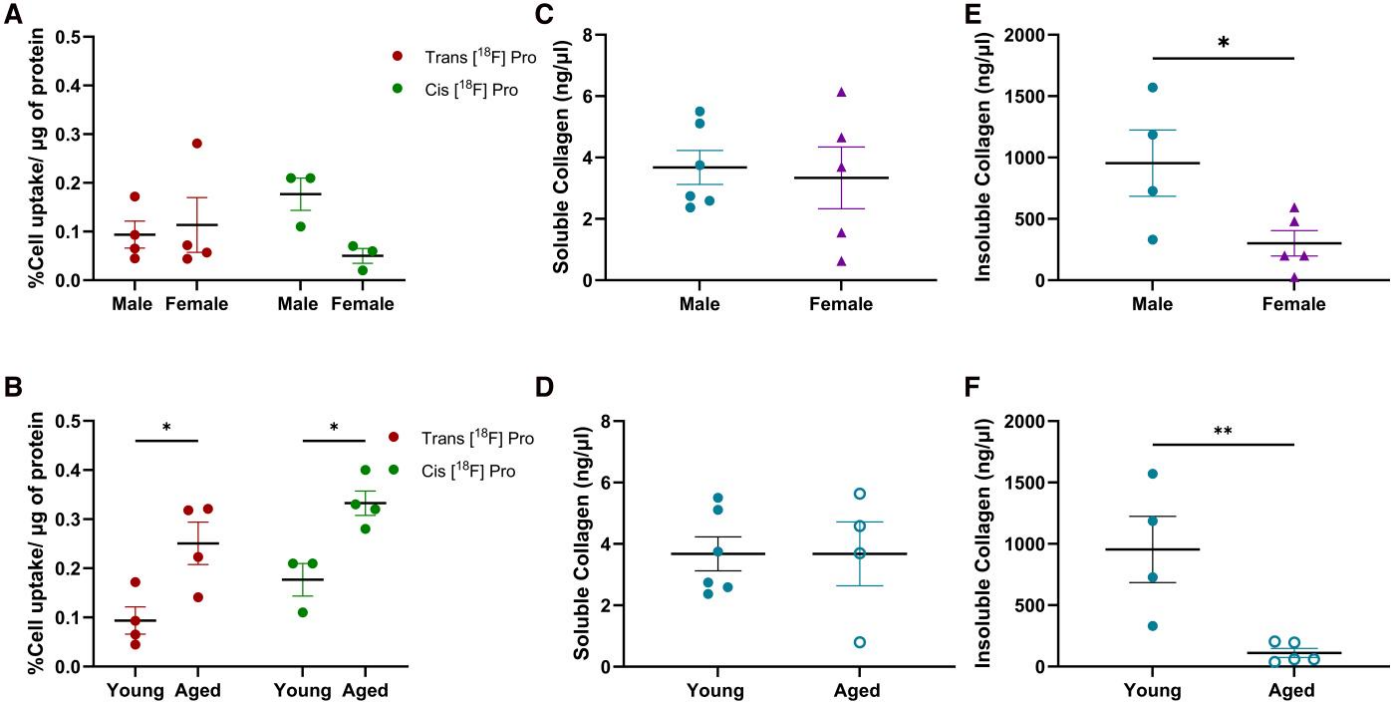
Effects of age and sex on collagen synthesis and deposition in primary rat cFbs. (*A–B*) Unhydroxylated and hydroxylated collagen synthesis measured by *cis*-/*trans*-4-[^18^F]fluoro-L-proline uptake in young male vs. female (A; *n* = 3–4) and young vs. aged male (B; *n* = 3–4) cFbs. (*C–D*) Soluble collagen content in the same groups (C; *n* = 5–6, D; *n* = 4–6). (*E–F*) Insoluble collagen deposition (E; *n* = 4–5, F; *n* = 4–5). Data are presented as mean ± SEM with individual points. Normality was assessed using the Shapiro–Wilk test, and comparisons were performed using unpaired *t*- or Mann–Whitney *U* tests as appropriate (*P* < 0.05, *P* < 0.01).

Quantification of *trans-/cis*-4-[^18^F]fluoro-L-proline demonstrated no significant sex differences in young cFbs (*Figure 1A*). In male cFbs, ageing increased in *cis-*4-[^18^F]fluoro-L-proline uptake (*P* = 0.02), suggesting upregulated interstitial collagen precursor synthesis (*Figure 1B*). Hydroxylated collagen synthesis [*trans*-4-(^18^F)fluoro-L-proline uptake], was also elevated in aged male cFbs relative (*P* = 0.04), (*Figure 1B*). Therefore, collagen synthesis was primarily age-dependent *in vitro*.

### 3.2 Collagen deposition

Soluble collagen content remained unaffected by either age or sex (*Figure 1C, D*). Insoluble collagen content—reflecting mature ECM— was greater in young males vs. females (*P* = 0.04) (*Figure 1 E*). However, a significant decline in insoluble collagen was observed with ageing in males (*P* = 0.02), despite increased precursor synthesis (*Figure 1F*). This dissociation suggests impaired collagen maturation or crosslinking in aged cFbs, but this remains a hypothesis that requires further biochemical validation.

## 4. Discussion

Ageing increased unhydroxylated and hydroxylated collagen synthesis but reduced insoluble collagen deposition in male fibroblasts. We hypothesize this uncoupling reflects age-related impairments in collagen maturation and ECM organization, potentially due to dysregulated post-translational modifications or enzymes such as lysyl oxidase (LOX) and matrix metalloproteinases.^2^ Further molecular assays are needed to confirm mechanisms, as such dysregulation may weaken collagen networks and impair ECM remodelling in the ageing heart.^2,7^ While *cis-/trans*-4-[^18^F]fluoro-L-proline served as synthesis proxies, direct pathway analyses (e.g. crosslinking, hydroxyproline) would clarify stage-specific effects.

No sex differences appeared in young fibroblasts’ synthesis, but males showed greater insoluble collagen accumulation—aligning with male-predominant adverse remodelling.^2,8^ We hypothesize the lack of a sex effect on synthesis reflects (i) regulation by sex hormones—absent *in vitro*—rather than intrinsic sex-linked pathways and (ii) the absence of aged female samples and small sample size which may have obscured sex effects. Future studies should include hormone supplementation and aged female fibroblasts to clarify sex–age interactions. Additionally, primary fibroblast cultures tend towards heterogeneous myofibroblast-like activation rather than a quiescent *in vivo* phenotype which may alter our findings.

From a translational perspective, these findings are relevant to heart failure—particularly HFpEF—where myocardial fibrosis drives diastolic dysfunction. Defining the cellular contributors to fibrosis, and how ageing and sex influence collagen handling, may help identify subgroups at risk of maladaptive ECM remodelling.

The observed dissociation between collagen synthesis and deposition in aged fibroblasts may explain the paradox in older patients, in whom myocardial stiffness and fibrosis increase without a clear rise in collagen gene expression.^9^ Our data points to impaired post-synthetic collagen maturation as a central mechanism in age-related fibrosis, consistent with reports of altered LOX-mediated crosslinking in aged mice.^10^ However, further *in vivo* studies are needed, *as in vitro* fibroblast models do not fully recapitulate physiological ageing.

## 5. Conclusion

This study shows that ageing significantly alters collagen metabolism in cFbs *in vitro*, increasing collagen synthesis while impairing deposition—particularly in male cells. Young male fibroblasts displayed greater insoluble collagen deposition than females, aligning with clinically observed sex-specific remodelling patterns. Elevated metabolic activity in aged fibroblasts further indicates an age-associated phenotypic shift. Together, these findings provide preliminary mechanistic insight into myocardial fibrosis and suggest that fibroblast-intrinsic changes contribute to sex- and age-related differences in cardiac remodelling. Future work should incorporate aged female fibroblasts and *in vivo* models to better define these pathways and validate therapeutic targets.

## Data Availability

Data available on request.
